# Application of dual *Nr4a1*-GFP *Nr4a3*-Tocky reporter mice to study T cell receptor signaling by flow cytometry

**DOI:** 10.1016/j.xpro.2020.100284

**Published:** 2021-01-21

**Authors:** Emma K. Jennings, David A.J. Lecky, Masahiro Ono, David Bending

**Affiliations:** 1 Institute of Immunology and Immunotherapy, College of Medical and Dental Sciences, University of Birmingham, Birmingham B15 2TT, UK; 2 Department of Life Sciences, Imperial College London, London SW7 2AZ, UK

**Keywords:** Flow Cytometry/Mass Cytometry, Immunology, Model Organisms

## Abstract

This protocol uses *Nr4a1*-GFP *Nr4a3*-Tocky mice to study T cell receptor (TCR) signaling using flow cytometry. It identifies the optimal mouse transgenic status and fluorochromes compatible with the dual reporter. This protocol has applications in TCR signaling, and we outline how to obtain high-quality datasets. It is not compatible with cellular fixation, and cells should be analyzed immediately after staining.

For complete details on the use and execution of this protocol, please refer to Jennings et al., 2020.

## Highlights

• Protocol uses *Nr4a1*-GFP and *Nr4a3*-Tocky mice to study TCR signal duration
• Optimized for use with homozygous *Nr4a3*-Tocky mice
• Guidance on fluorochrome choices compatible with GFP and Timer-Blue and Timer-Red
• Use of an open “dump” channel on flow cytometer can improve signal to noise ratio

## Graphical Abstract

**Figure:**
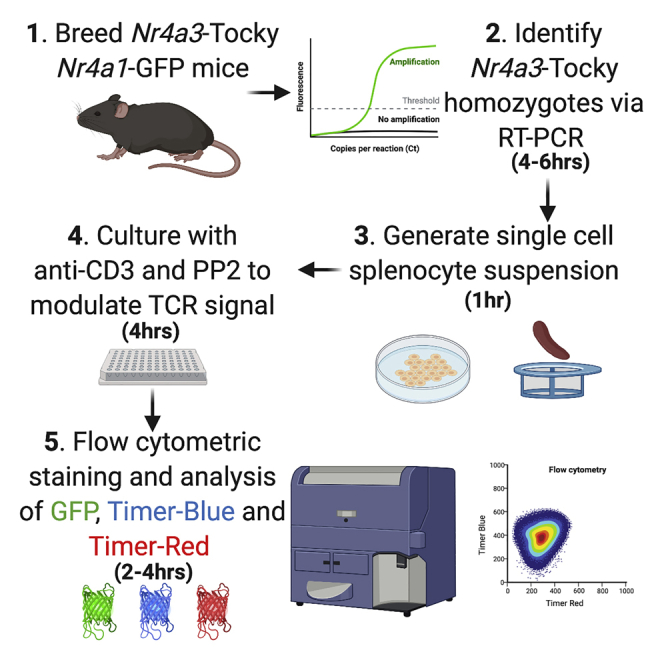

## Before you begin

### Determine zygosity of *Nr4a3*-Tocky mice

*Nr4a1*-GFP (Moran et al., 2011) and *Nr4a3*-Tocky (Bending et al., 2018b) are Bacterial Artificial Chromosome (BAC) transgenic mice. The protocol is optimized for use of “homozygous” *Nr4a3*-Tocky BAC and “heterozygous” *Nr4a1*-GFP mice.

**CRITICAL:** Use of heterozygous *Nr4a3*-Tocky in combination with *Nr4a1*-GFP leads to reduced expression of Nr4a3-Timer (Jennings et al., 2020). Therefore, it is strongly recommended that *Nr4a3*-Tocky homozygous mice are used when these are mated to *Nr4a1*-GFP mice.

***Note:*** The following primers are made up to 100 μM final concentration as master stocks. From these 1 in 10 dilutions of working 10 μM stocks are made.

**Table:** 

Primer	Sequence
*Timer BAC* F	CGCGGAACTAACTTCCCCTC
*Timer BAC* R	GTCTTGACCTCAGCGTCGTA
*Il2ra* F	CAGGAGTTTCCTAAGCAACG
*Il2ra* R	CTGTGTCTGTATGACCCACC

***Note:****Timer BAC* F+R produce a PCR product of approximately 180 bp; *Il2ra* F+R product is approximately 200 bp.

**Timing: 2–6 h (DNA extraction typically takes 4 h, RT-PCR 1.5 h with 30 min preparation)**

1. DNA extraction from ear tissue biopsy using PureLink Genomic DNA mini Kit (Thermo Fisher) according to manufacturer’s instructions.

2. Preparation of real-time (RT)-PCR reaction using PowerUP SYBR green and primers designed for the *Timer BAC* transgene and an endogenous *Il2ra* control gene.
  a. Prepare reaction mix for *Timer BAC* and genomic *Il2ra.*

**Table:** 

Reagent	Final concentration (mM or μM)	Amount
2× PowerUP SYBR Green	1×	5 μL
10 μM forward primer	250 nM	0.25 μL
10 μM reverse primer	250 nM	0.25 μL
ddH_2_O	n/a	3.5 μL
DNA template	n/a	1 μL
**Total**	**n/a**	**10 μL**
  b. Pipette 9 μL of reaction mix onto the wells of a 384 well PCR plate (or 96 well plate for compatible machines) in technical triplicates.
  c. Add 1 μL of template DNA (concentration 10–50 ng/μL) to each reaction.
  d. Seal the plate with optical adhesive and centrifuge for 15 s at 500 × *g*.
  e. Set up real-time PCR on ABI 7900HT machine (or other SYBR green compatible machine) with the following cycling conditions:
    55°C 2 min
    95°C 2 min
    35 cycles of the following three steps:
    95°C 15 s
    60°C 30 s
    72°C 30 s
  f. Confirm reaction specificity by melt curve analysis – single peak should be observable.
  g. Set same threshold of 0.2 for both *Il2ra* and *BAC Timer* to calculate Ct.
  h. Calculate delta (Δ)Ct using the formula:
    ΔCt=Ct (*Il2ra*)-Ct(*BAC Timer*)
    ***Note:*** Depending on machine and SYBR green master mix, the ΔCt may vary. Controls with known zygosity should be used to identify ΔCt ranges.
  i. Figure 1 shows typical ΔCt using this protocol and ABI 7900HT machine.

**Figure 1:**
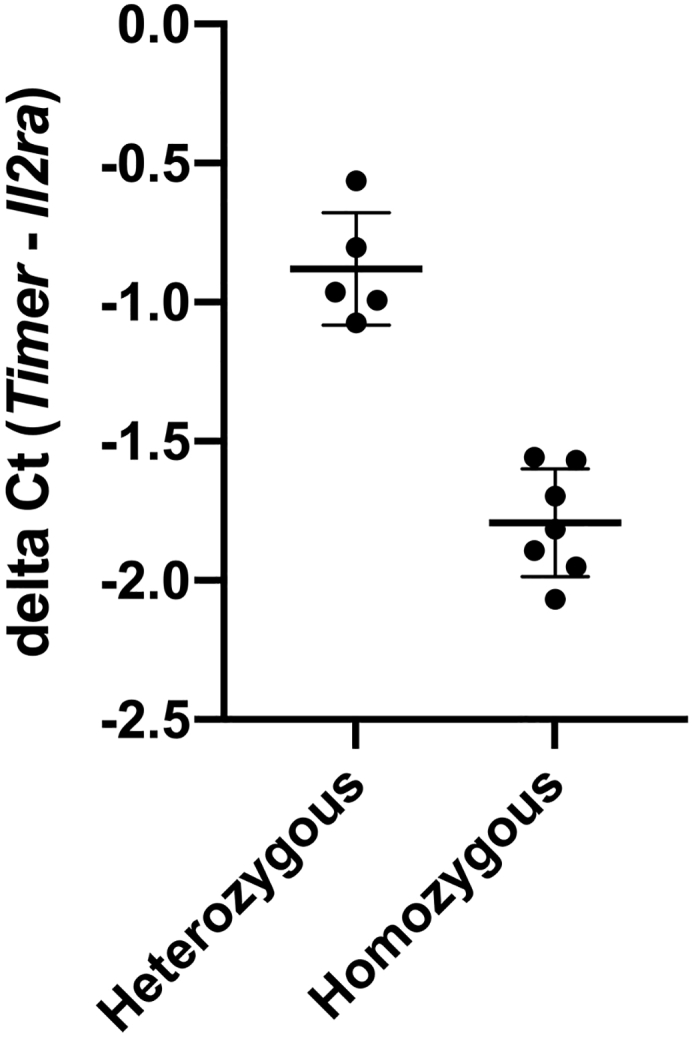
Typical delta (Δ)Ct values for homozygote and heterozygote *Nr4a3*-Tocky mice ΔCt values based on Ct for *Timer Bac* – Ct for *Il2ra* for heterozygote (n = 5) and homozygote (n = 7) *Nr4a3*-Tocky mice. Dots represent individual mice and bars represent mean ± SEM.
  j. Determine zygosity of line and use *Nr4a3*-Tocky homozygous in combination with *Nr4a1*-GFP for experiment.

### Panel design

**Timing: 3–4 h**

**CRITICAL:** Key to high-quality analysis is optimal panel design and this should be validated before undertaking experiments. Here we illustrate the compensation matrix and optimized fluorochrome combinations using a BD LSR Fortessa X-20 machine. It is by no means exhaustive but represents a good starting point for panel design.

3. GFP, Timer-Blue, and Timer-Red are excitable by different lasers and detected by different filters (see Materials and equipment). The assumption is that Timer-Blue and Timer-Red do not spectrally overlap (Subach et al., 2009), and therefore no compensation is required between these two channels (Bending et al., 2018b). Single color controls can be generated through using a *Nr4a1*-GFP line (or equivalent GFP expressing cell line), and *Nr4a3*-Tocky cells. To generate a single Blue and Red color control:
  a. Place 1 million *Nr4a3*-Tocky splenocytes into a 96 well round bottom plate containing 10% FBS RPMI (v/v) and stimulate with 5 μg/mL soluble anti-CD3 for 4 h.
    ***Note:*** Nr4a3-Blue expression will remain for long as as a TCR stimulus is applied in vitro; however, the transition of Blue to Red has a half-life of approximately 4 h, meaning stimulus for longer periods will increase Red fluorescence of cells.
  b. Harvest cells and maintain on ice until analysis on flow cytometer, gate on Nr4a3-Blue^+^ (BV421 channel) and Nr4a3-Red^−^ (PE-Texas Red channel) cells to use as a single color Blue control.
  c. Use unstimulated Nr4a3-Tocky T cells and gate on Nr4a3-Blue^−^Nr4a3-Red^+^ to generate a single Red color control.
    ***Note:***Figure 2 shows that Blue, Red and GFP colors show no overlap (see also Table 1)
    **Figure 2:**
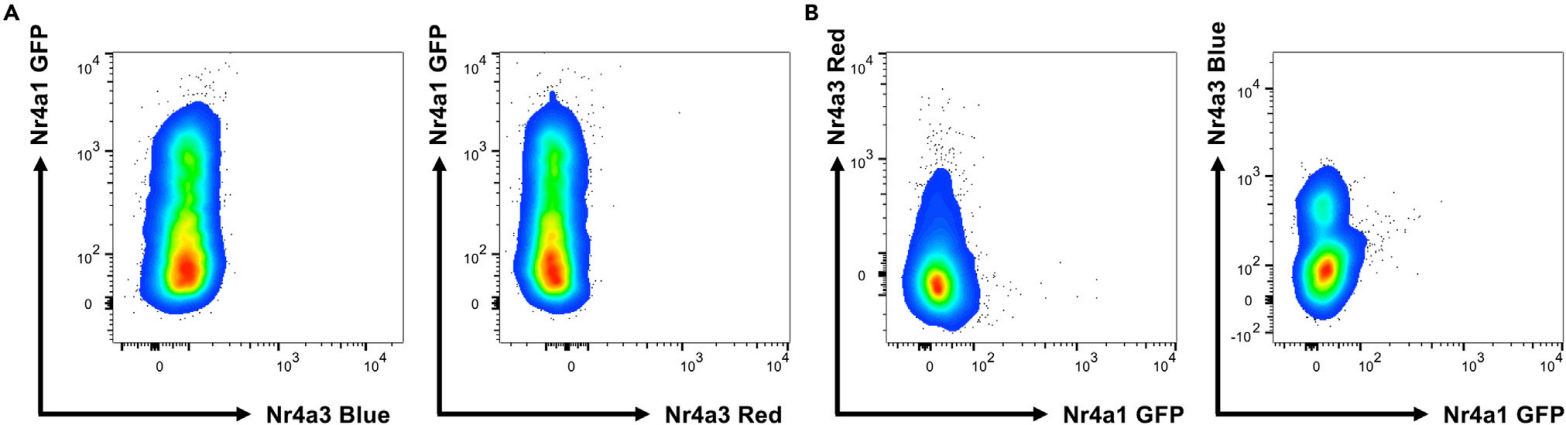
GFP, Timer-Blue, and Timer-Red show no spectral overlap (A) Thymocytes from OTI *Nr4a3*-Tocky *Nr4a1*-GFP mice (which do not express Nr4a3-Timer in the thymus; Jennings et al., 2020) were analyzed for expression of Nr4a3-Blue versus Nr4a1-GFP and Nr4a3-Red versus Nr4a1-GFP channels. (B) Splenocytes from *Nr4a3*-Tocky (pure) line were analyzed for Nr4a1-GFP versus Nr4a3-Red (left) or stimulated for 3 h with anti-CD3 and then Nr4a3-Blue^+^Nr4a3-Red^−^ cells analyzed for Nr4a1-GFP versus Nr4a3-Blue. These single color controls were used to make the compensation matrix in Table 1.

**Table 1:** Compensation matrix for GFP, Timer-Blue, Timer-Red, and tested channels on the BD LSR FORTESSA X-20

	GFP	Blue	Red	APC-A	Alexa 700-A	APC-Cy7-A	PerCP-Cy5-5-A	BUV737-A	BUV395-A	PE-Cy7-A	BV605-A	BV711-A	PE-A
GFP		0.00%	0.00%	0.00%	0.00%	0.00%	1.00%	0.00%	0.00%	0.00%	0.00%	0.00%	0.00%
Blue	0.00%		0.00%	0.00%	0.00%	0.00%	0.00%	0.00%	0.00%	0.00%	8.00%	0.00%	0.00%
Red	0.00%	0.00%		0.00%	0.00%	0.00%	4.00%	0.00%	0.00%	2.20%	0.00%	0.00%	16.00%
APC-A	0.00%	0.00%	0.90%		40.00%	11.00%	2.00%	13.00%	0.00%	3.40%	0.00%	4.60%	0.00%
Alexa 700-A	0.00%	0.00%	0.00%	3.30%		27.00%	8.00%	28.50%	0.00%	5.50%	0.00%	11.00%	0.00%
APC-Cy7-A	0.00%	0.00%	0.60%	1.10%	5.50%		0.30%	12.00%	0.00%	18.00%	0.00%	0.40%	0.20%
PerCP-Cy5-5-A	0.00%	0.00%	0.00%	7.00%	9.50%	3.30%		25.00%	0.00%	4.00%	0.00%	29.00%	0.00%
BUV737-A	0.00%	0.00%	0.00%	0.10%	3.40%	1.70%	5.30%		0.95%	0.20%	0.00%	0.80%	0.00%
BUV395-A	0.00%	0.20%	0.00%	0.00%	0.00%	0.00%	0.00%	0.00%		0.00%	0.00%	0.00%	0.00%
PE-Cy7-A	0.10%	0.00%	3.50%	0.00%	0.30%	5.80%	5.20%	5.00%	0.00%		0.00%	0.00%	2.20%
BV605-A	0.00%	3.20%	46.00%	0.00%	0.00%	0.00%	1.40%	9.10%	0.20%	0.70%		11.70%	8.30%
BV711-A	0.00%	9.40%	0.00%	1.00%	14.00%	3.60%	25.00%	84.00%	0.00%	0.00%	0.60%		0.00%
PE-A	0.40%	0.00%	135.00%	0.00%	0.00%	0.00%	12.00%	0.00%	0.00%	0.20%	8.00%	0.00%	

Compensation matrix generated on FlowJo software indicating percentage spectral overlap between various channels. Single color controls were generated using activated splenocytes from pure *Nr4a3*-Tocky mice (for Timer-Blue and Timer-Red; Figure 2), or thymus from OTI *Nr4a1*-GFP *Nr4a3*-Tocky (for Nr4a1-GFP single color; Figure 2). Non-transgenic cells were stained with the following antibodies to generate single color controls: anti-mouse CD5 APC; viability dye eFluor780 (APC-Cy7); anti-mouse TCRbeta PerCP-Cy5.5; anti-mouse CD8 BUV737; anti-mouse CD4 BUV395; anti-mouse CD8 PE-Cy7; anti-mouse-CX3CR1 BV605; anti-mouse CD11c BV711; anti-mouse CD103 PE.

4. For panel design it is recommended to use a viability dye (such as eFluor780) to exclude dead cells. A typical compensation matrix involving commonly used channels is shown (Table 1). Typical compensation requirements between GFP, Timer-Blue, and Timer-Red with other channels are bold highlighted. Troubleshooting 1

***Note:*** Caution should be used when using BV711 and BV605, and PE-Cy7 due to the spectral overlaps between them and Timer-Red and Timer-Blue channels. We recommend that single color compensation controls for all tandem dyes use the dye that is in the final panel. We advise not using PE or BV510 due to the close proximity to Nr4a3-Red and Nr4a3-Blue. FITC is not compatible with GFP.

## Key resources table

**Table:** 

REAGENT or RESOURCE	SOURCE	IDENTIFIER
**Antibodies**		

Anti CD3ϵ Armenian hamster monoclonal 145-2C11	BioLegend	Cat# 100301; RRID: AB_312666
Anti-CD4 BUV737 rat monoclonal GK1.5	BD Biosciences	Cat# 564298; RRID: AB_2738734
Anti CD8 BUV395 rat monoclonal 53-6.7	BD Biosciences	Cat# 563786; RRID: AB_2732919
Anti CD69 AF700 Armenian hamster monoclonal H1.2F3	BioLegend	Cat# 104539; RRID: AB_2566304
Anti CD103 PE Armenian hamster monoclonal 2E7	BioLegend	Cat# 121406; RRID: AB_535948
Anti CX3CR1 BV605 mouse monoclonal SA011F11	BioLegend	Cat# 149027; RRID: AB_2565937
Anti CD11c BV711 Armenian hamster monoclonal N418	BioLegend	Cat# 117349; RRID: AB_2563905
Anti CD8 PE-cy7 rat monoclonal 53-6.7	BioLegend	Cat# 100722; RRID: AB_312760
Anti CD5 APC rat monoclonal 52-7.3	BioLegend	Cat# 100625; RRID: AB_2563928
Anti-TCRbeta PerCP-Cy5.5 Armenian hamster monoclonal H57-597	Tonbo Biosciences	Cat# 65-5961-UO25; RRID: AB_2621911

**Chemicals, peptides, and recombinant proteins**		

PP2	Sigma-Aldrich	Cat# P0042; CAS: 172889-27-9
RPMI 1640 (referred to as RPMI)	Gibco	21875034
Fetal bovine serum (FBS)	Gibco	10500064
PBS	Sigma-Aldrich	D8537

**Critical commercial assays**		

Applied Biosystems SYBR green power up master mix	Thermo Fisher	Cat# A25752
eFluor-780 fixable viability dye	eBioscience	Cat# 65-0865-14
PureLink genomic DNA mini kit	Thermo Fisher	K182002
Red blood cell lysis buffer	Thermo Fisher	00-4333-57

**Experimental models: organisms/strains**		

Mouse: *Nr4a3*-Tocky founder line 323	Dr. Masahiro Ono, Imperial College London	PMID: 29941474 (Bending et al., 2018b)
Mouse: Nr4a1/Nur77 GFP	Jackson Laboratory	Strain code: 016617
Mouse: OTI	Charles River Laboratories	Strain Code: 642

**Oligonucleotides**		

*Timer BAC* F*:* CGCGGAACTAACTTCCCCTC	Sigma-Aldrich	This paper
*Timer BAC* R*: GTCTTGACCTCAGCGTCGTA*	Sigma-Aldrich	This paper
*Il2ra* F*: CAGGAGTTTCCTAAGCAACG*	Sigma-Aldrich	This paper
*Il2ra* R*:* CTGTGTCTGTATGACCCACC	Sigma-Aldrich	This paper

**Software and algorithms**		

GraphPad Prism 8 and 9	GraphPad	https://www.graphpad.com/scientific-software/prism/
FlowJo v10.5.3	FlowJo	https://www.flowjo.com/solutions/flowjo

**Other**		

BD LSR FORTESSA X-20 machine	BD Biosciences	Custom machine
ABI 7900HT	Thermo Fisher	Custom machine

## Materials and equipment

### Mice

All animal experiments were performed in accordance with local Animal Welfare and Ethical Review Body at the University of Birmingham and under the authority of a Home Office project license, P18A892E0A held by D.B. Animals were housed in specific pathogen-free conditions with appropriate housing conditions and husbandry as specified by NC3Rs. *Nr4a3*-Tocky mice expressing a BAC containing FT Fast mCherry mutant (Subach et al., 2009) under the influence of *Nr4a3* regulatory regions on the C57BL/6J background as previously described (Bending et al., 2018a; Bending et al., 2018b) were bred to Nr4a1/Nur77-GFP mice expressing a BAC containing *GFP* transgene under the influence of *Nr4a1* regulatory regions on C57BL/6J background as previously described (Moran et al., 2011). These were mated to OTI mice (expressing the TCR (Vα2, Vβ5) of the OVA257-264-specific CTL clone 149.42) to generate OTI *Nr4a3*-Tocky *Nr4a1*-GFP mice.

### Flow cytometer – BD LSR FORTESSA X-20

Data were acquired on a BD LSR FORTESSA X-20 flow cytometer, with the configuration detailed below. Dyes require careful compensation considerations are marked with an asterisk. Other cytometers with similar setups would also be suitable.

**Table:** 

BD LSR FORTESSA X-20 dyes tested		
Laser	Filter	Tested dye(s)
355 nm (**UV**)	740/35	BUV737
	379/28	BUV395
405 nm (**violet**)	450/50	Timer-Blue
	525/50	**not advised BV510**
	610/20	BV605∗
	670/30	not tested
	710/50	BV711∗
	780/60	not tested
488 nm (**blue**)	530/30	GFP
	670/30	PerCP-Cy5.5 (or DUMP)
561 nm (**yellow**)	586/15	**not advised PE**
	610/20	Timer-Red
	670/30	not tested
	710/50	not tested
	795/71	PE-Cy7∗
642 nm (**red**)	671/30	APC
	722/44	AF700
	795/70	efluor780

**Table:** BD LSR FORTESSA X-20 settings

Parameter	Laser	Filter	Gain	Voltage
BUV395-A	355 nm (UV)	379/28	1	576
BUV737-A	355 nm (UV)	740/35	1	750
BV421-A	405 nm (violet)	450/50	1	406
BV605-A	405 nm (violet)	610/20	1	549
BV711-A	405 nm (violet)	710/50	1	514
FITC-A	488 nm (blue)	530/30	1	405
PerCP-Cy5-5-A	488 nm (blue)	670/30	1	583
PE-A	561 nm (yellow)	586/15	1	503
PE-Texas Red-A	561 nm (yellow)	610/20	1	612
PE-Cy7-A	561 nm (yellow)	795/71	1	500
APC-A	642 nm (red)	671/30	1	520
APC-Alexa 700-A	642 nm (red)	722/44	1	487
APC-Cy7-A	642 nm (red)	795/70	1	493

***Alternatives:*** Alternative fluorochrome formats are available for many channels, however these should be tested in advance for compatibility to Nr4a3-Blue, Nr4a3-Red, and Nr4a1-GFP. Alternative flow cytometer settings and filters are also available, but each user should optimize voltages and panel design to their own machines.

### Buffers

Staining and washing buffers: consisted of 1× PBS supplemented with 2% FBS (v/v).

Culture media: 10% FBS (v/v) RMPI 1640 was supplemented with 1% penicillin/streptomycin (v/v, made from 100× stock at 5,000 units/mL penicillin, 5,000 μg/mL streptomycin, Life Technologies).

## Step-by-step method details

### Assessing T cell receptor signal duration in the regulation of *Nr4a1* and *Nr4a3* expression using *Nr4a1*-GFP and *Nr4a3*-Tocky dual reporter mice

#### Generation of single-cell splenocyte suspension

**Timing: 1–2 h**

This part generates a single-cell suspension of splenocytes for use in experimental cultures.

1. Harvest spleen from *Nr4a1*-GFP *Nr4a3*-Tocky mice (Jennings et al., 2020) in TC hood.
  a. Mice are euthanized by cervical dislocation, and death confirmed by cessation of circulation.
  b. Spleen is removed, ensuring complete detachment of the pancreas and placed into a 1.5 mL Eppendorf containing 0.5 mL 10% FBS RPMI.
2. Generation of single-cell suspension in tissue culture hood.
  a. Into a sterile 5 mL petri dish, place a 70 μm cell strainer and add the spleen and 1 mL of 10% FBS RPMI buffer. (Alternatively, a strainer can be placed in a 50 mL Falcon tube for processing).
  b. Using the syringe plunger from a 5 mL syringe, gently force the spleen through the cell strainer.
  c. Using a P1000 pipette add 1 mL 10% FBS RPMI buffer to wash the strainer.
  d. Filter the splenocyte solution back through the strainer by taking from suspension in the petri dish and pipetting back onto the strainer. Repeat this 8–10 times using P1000 pipette until suspension is homogenous.
  e. Pool the splenocyte suspension by transferring it into a 15 mL Falcon tube.
  f. Wash strainer with 1 mL 10% FBS RMPI and transfer this to the 15 mL Falcon tube.
  g. Centrifuge Falcon tube at 500 × *g* for 5 min at 15°C–20°C.
  h. Decant supernatant and proceed to perform red blood cell lysis by resuspending the cell pellet in 1 mL of RBC lysis buffer.
  i. Incubate for 2 min on ice.
  j. Top up the 15 mL Falcon tube with 9 mL 10% FBS RPMI and centrifuge at 500 × *g* for 5 min at 15°C–20°C.
  k. Decant supernatant and resuspend cell pellet at cell concentration of 5 million/mL in 10% FBS RPMI and keep cells on ice.

#### Culture setup

**Timing: 0–4 h**

This part established how to modulate the TCR signal length in splenocyte cultures from *Nr4a1*-GFP *Nr4a3*-Tocky mice.

***Optional:*** Cultures are set up on 96 well round bottom plates (Corning) in a final volume of 190 μL, with 10 μL volume used to add the PP2 inhibitor, however depending on cell numbers and desired interactions other plate formats may also be suitable.

***Note:*** PP2 is reconstituted in sterile DMSO to a master stock concentration of 20 mM.

3. Prepare master mixes of anti-CD3 and PP2, a src-kinase family inhibitor which will pharmacologically terminate TCR signaling in culture.
  a. 20 μg/mL anti-CD3, (pre-warmed to 37°C).
  b. 200 μm PP2 (keep on ice).
4. Pipette 100 μL of splenocyte suspension which is at 5 million/mL into 8 wells of 96 well round bottom plate. For this experiment there are 8 conditions representing 0, 2.5, 5, 15, 30, 60, 120, and 240 min of TCR stimulation.
5. Add 40 μL of 10% FBS RPMI to each well.
6. Spin plate for 1 min at 100 × *g* to settle cells to bottom of plate.
7. Place plate in incubator for 30 min to equilibrate at 37°C.
8. Remove plate and add 50 μL of 20 μg/mL anti-CD3 stock (final concentration 5 μg/mL) to wells.
9. Add 10 μL of 200 μM PP2 to 0-min stimulation (gives final concentration 10 μM).
10. Place plate in incubator with 5% CO_2_ and 37°C and start timer.
11. At 2.5-, 5-, 15-, 30-, 60-, 120-min remove plate and add 10 μL of 200 μM PP2 inhibitor. Place plate back in incubator until 240 min has expired.

***Note:*** During incubation, the staining mix can be prepared (see below).

12. After 240 min proceed immediately to staining.

#### Staining for flow cytometric analysis

**Timing: 0.5–1 h**

13. Centrifuge culture plate at 500 × *g* for 3 min, flick plate to decant supernatant and while still inverted blot against paper towel.
14. Add 25 μL of staining mix prepared as below in 2% FBS PBS (v/v). In our hands viability dye staining can be performed simultaneously with surface staining without adversely affecting performance.

***Note:*** Include single color compensation controls in addition to GFP, Timer-Blue, and Timer-Red.

**Table:** 

Stain	Stain dilution
Viability Dye eFluor780	1 in 2,000
CD4 BUV395	1 in 200
CD8 BUV737	1 in 200
CD69 AF700	1 in 50
*Optional* PEcy7	*Optional stain*
*Optional* APC	*Optional stain*
*Optional* PerCP-Cy5.5	*Optional stain / DUMP*

15. Incubate for 20 min at 4°C.
16. Add 180 μL of wash buffer to each well and centrifuge plate at 500 × *g* for 3 min, flick plate to decant supernatant and while still inverted blot against paper towel.
17. Resuspend pellet in 150 μL 2% FBS PBS and transfer to 5 mL FACS tube (BD Biosciences). Wash well with 150 μL 2% FBS PBS and transfer to FACS tube to make 300 μL final volume.
18. Place on ice in the dark and acquire samples on the flow cytometer within 2 h of staining.

**CRITICAL:** Cells should be analyzed as soon as possible following staining. In our hands, slow maturation of Blue to Red can occur after prolonged periods (e.g., overnight) at 4°C.

## Expected outcomes

Using this protocol, it is expected to see an early increase in Nr4a1-GFP expression commencing within as little as 5–10 min of stimulation time. Troubleshooting 2Nr4a3-Blue expression will start to increase after 30–60 min of stimulation. After 12 min of TCR signaling 50% of T cells should be Nr4a1-GFP^+^. After 70 min of TCR stimulation 50% of T cells should be Nr4a3-Blue^+^. With 4 h of TCR signaling >85%–90% of CD4^+^ and CD8^+^ T cells should be Nr4a1-GFP^+^Nr4a3-Blue^+^ (see Figure 4D in (Jennings et al., 2020)) Troubleshooting 4

## Quantification and statistical analysis

To determine the percentage positivity, a non-transgenic line can be used to set the threshold for Nr4a1-GFP and Nr4a3-Blue positivity. Given that Nr4a1-GFP has higher background expression it is important that the data are normalized to the minimum at T=0 min and maximum at T=240 min using the following formulas:

$$\text{Nr}4\text{a}1-\text{GFP Normalized}=\left(\frac{\left(\%\text{Nr}4\text{a}1-\text{GFP in condition}\right)-\left(\text{\%Nr}4\text{a}1-\text{GFP at T}=0\right)}{\left(\text{\%Nr}4\text{a}1-\text{GFP at T}=240\right)-\left(\text{\%Nr}4\text{a}1-\text{GFP at T}=0\right)}\right)\times 100$$

$$\text{Nr}4\text{a}3-\text{Blue Normalized}=\left(\frac{\left(\%\text{Nr}4\text{a}3-\text{Blue in condition}\right)-\left(\text{\%Nr}4\text{a}3-\text{Blue at T}=0\right)}{\left(\text{\%Nr}4\text{a}3-\text{Blue at T}=240\right)-\left(\text{\%Nr}4\text{a}3-\text{Blue at T}=0\right)}\right)\times 100$$

## Limitations

This protocol is designed for the analysis of Nr4a1-GFP and Nr4a3-Timer expression patterns by flow cytometry. Other fluorescence-based analysis techniques such as confocal microscopy have not been tested or validated on the dual reporter. This protocol also relies on the analysis occurring within 2 h of staining, and samples being kept on ice in the dark to prevent the maturation of Timer-Blue into Timer-Red. This protocol is also not compatible with fixation and intranuclear factor staining with kits such as the eBioscience Foxp3 / Transcription Factor Staining Buffer Set.

## Troubleshooting

### Problem 1

A diagonal population of Blue^+^Red^+^ cells appear in unstimulated controls.

### Potential solution

**Figure 3:**
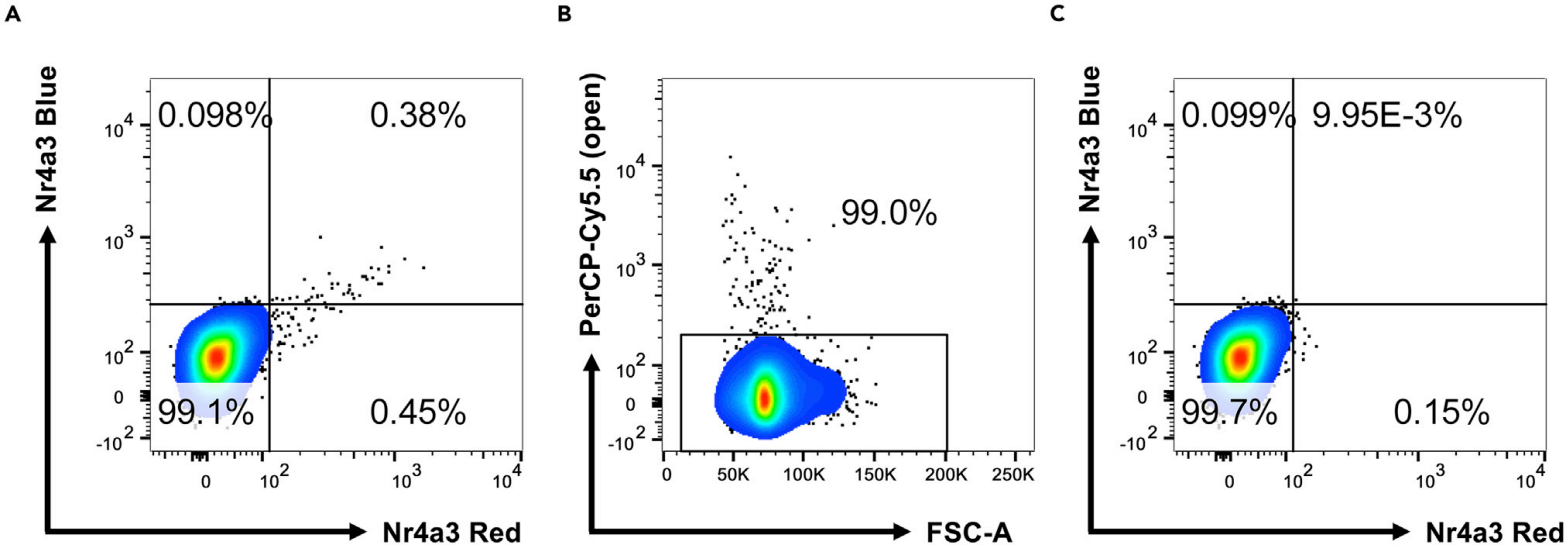
Use of PerCP-Cy5.5 channel as a dump to reduce noise (A) Lymphocytes from wild-type mice displaying autofluorescence in the Timer-Blue versus Timer-Red channels, which appears as a diagonal line. (B) By gating on FSC-A versus PerCP-Cy5.5 (open channel no stain), the noise can be significantly reduced by excluding PerCP-Cy5.5^+^ cells. (C) The result is that lymphocytes from wild-type mice gated as PerCP-Cy5.5^−^ display very low noise in the Timer-Blue versus Timer-Red channels.

This is typically seen in preparations from tissues that have increased autofluorescence of some cells. To reduce background, we advise having a “dump” channel open such as the PerCP-Cy5.5 channel. If no dye is used in this channel, background noise is reduced substantially (Figure 3).

### Problem 2

Weak signal in Blue channel but strong signal in GFP after stimulation.

### Potential solution

This is typically caused by using *Nr4a3*-Tocky heterozygous BAC mice in combination with *Nr4a1*-GFP BAC mice. While *Nr4a3*-Tocky heterozygous mice are usable alone, when crossed with *Nr4a1*-GFP BAC mice the Nr4a3-Timer signal is quenched. We advise checking zygosity of line and repeating experiment with a confirmed *Nr4a3*-Tocky homozygote with *Nr4a1*-GFP heterozygote.

### Problem 3

Ex vivo Timer-Red signal is dim for single color staining control.

### Potential solution

Splenocytes can be activated with soluble anti-CD3 for 4 h before washing and keeping in fridge for 24 h. The Blue proteins will mature to the terminal Red form and provide a brighter single Red color control.

### Problem 4

Cells show a clear Blue^+^Red^+^ pattern, even after a short 4-h stimulation.

### Potential solution

This may occur if cells are not analyzed immediately. The half-life of the timer protein is 4 h, and this process can be stopped by keeping cells on ice before acquisition. We strongly recommend analysis within 2 h of staining of samples. Overnight incubation in the fridge is likely to reduce Timer-Blue and increase Timer-Red signal.

## Resource availability

### Lead contact

Further information and requests for resources and reagents should be directed to and will be fulfilled by the Lead Contact, David Bending (d.a.bending@bham.ac.uk ).

### Materials availability

*Nr4a3*-Tocky mice are available under MTA from Dr Masahiro Ono, (Imperial College London, UK). Nr4a1/Nur77-GFP mice are available from the Jax lab (strain number 016617).

### Data and code availability

All data from the study are present here or in Jennings et al., 2020.
